# Protective Effects of Natural Lipophilic Antioxidants on UV-Induced Lipid Oxidation in Liposomes and Their Enhancement

**DOI:** 10.3390/antiox14121450

**Published:** 2025-12-02

**Authors:** Anna Heidrich, Melvin Höfer, Volker Böhm

**Affiliations:** Institute of Nutritional Sciences, Friedrich Schiller University Jena, 07743 Jena, Germany; anna.heidrich@uni-jena.de (A.H.);

**Keywords:** antioxidant activity, carotenoids, tocopherol, vitamin C, trolox, lipid oxidation, antioxidant degradation, liposomes, UV radiation

## Abstract

Antioxidants, especially lipophilic antioxidants, absorb ultraviolet (UV) radiation and protect human skin from radicals that lead to oxidation reactions. The differences in the protective effects of carotenoids and α-tocopherol against UV radiation and the possible enhanced effects by the polar antioxidants, vitamin C and Trolox, need further investigation. Therefore, malondialdehyde was analyzed as a biomarker for lipid oxidation using the Thiobarbituric Acid-Assay (TBA-Assay) in liposomes irradiated with UV-C, UV-B, and UV-A radiation (254 nm, 320 nm, and 360 nm). In addition, antioxidant degradation was analyzed using HPLC with a diode array or fluorescence detector. The lipophilic antioxidants differ in their effect mainly due to their polarity and the associated different localization in the lipid bilayer. No pro-oxidative effect was observed at antioxidant concentrations close to saturation. The antioxidant effect was low at small concentrations, mainly due to aggregation of the antioxidants. The protective effect at higher antioxidant concentrations increased from up to 25–72% under UV-C, over 59–77% under UV-B, to 77–86% under UV-A radiation. Vitamin C proved to be 2–40 times less effective depending on the wavelength and the lipophilic antioxidant. Mixtures of lipophilic and hydrophilic antioxidants showed partially additive or synergistic effects. This appears to be dependent on concentration and ratio.

## 1. Introduction

**Figure 1:**
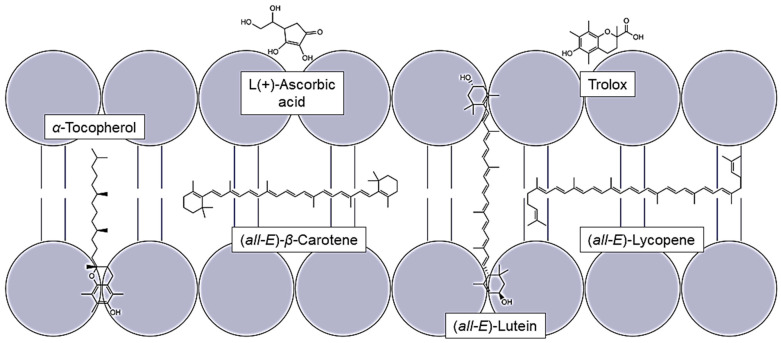
Localization of different lipophilic and hydrophilic antioxidants in the lipid bilayer.

Ultraviolet radiation, divided into UV-A (320–400 nm), UV-B (280–320 nm) and UV-C (200–280 nm), is classified by the International Agency for Research on Cancer (IARC) in Group I “carcinogenic to human” [1,2]. Eighty percent of the cutaneous melanoma cases worldwide are caused by UV radiation [3]. If the number of new melanoma cases remains the same until 2040, the incidence will increase by 50% [4]. UV-C radiation is filtered out by ozone but, due to its high energy, it is used in disinfection and other technical areas [5]. Humans are exposed to sunlight in their daily life. With only 0.1–0.4% UV-B and 3.9–4.4% UV-A radiation, it can still lead to erythema and ultimately to a sunburn and an increasing risk for melanocytic and non-melanocytic skin cancer [6,7,8]. One cause of the damages mentioned is the generation of free radicals and reactive oxygen species (ROS), like hydroxyl and peroxyl radicals, or singlet oxygen and secondary radicals, for example, lipid and lipid peroxyl radicals. Their concentration in specific skin layers is dependent on the wavelength, since the more energetic the radiation is, the less deep its penetration into the skin. In human or pig skin irradiated with UV-A, ROS, measured by electron spin resonance spectroscopy, can be found even in the dermis, with UV-B only in the epidermis and with UV-C in the stratum corneum, the first skin layer [9]. These free radicals are harmful to cells because they cause oxidized unsaturated fatty acids, resulting in a loss of fluidity and cell lysis, oxidized glycosides, influencing the activity of interleukins and the formations of prostaglandins, hormones, and neurotransmitters. They inactivate or denature proteins and modify DNA-bases, leading to mutagenesis and carcinogenesis [10]. To protect itself from radicals, the skin contains enzymes and antioxidants. Antioxidants are found in different concentrations and combinations in the skin and can be influenced by nutrition [11,12]. In particular, the lipophilic antioxidants *α*-tocopherol, mainly provided by vegetable oils, and several carotenoids, present in fruits and vegetables, were already analyzed in human studies, and a protective effect against UV radiation was found [13,14,15,16]. *α*-Tocopherol and carotenoids protect firstly by absorbing UV light, secondly by de-exiting photosensitizers, thirdly by reducing radicals, and lastly by re-reducing other antioxidants. Instead of other vitamin E derivatives, *α*-tocopherol was chosen because it is the most widely available and has the greatest potential for energy stabilization [17,18]. In addition, carotenoids can directly interact with genes or gene-regulatory signaling pathways as well [19]. Carotenoids in organic solutions quench singlet oxygen mainly by an electron transfer, producing a carotenoid in the triplet state and coming back to the ground state by heat dissipation [20,21]. Carotenoids also quench ROS by forming, mostly, endoperoxides. The quenching rate rises with a greater number of conjugated double bonds, such as lycopene, compared to carotenoids with less conjugated double bonds like lutein. The presence of cyclic structures in the carotenoid molecule causes a loss of planarity and thus an effectively shorter length of the π-electron system [22,23]. Zanfini et al., 2010, came to the same conclusion after analyzing antioxidant capacity using the TEAC test (Trolox Equivalent Antioxidative Capacity), reaching the following order: lycopene > *β*-carotene > lutein > *α*-tocopherol [24]. In comparison to carotenoids, *α*-tocopherol does not have a long system of conjugated double bonds, but its chromanol ring leads to similar protective effects since the phenolic hydrogen atom can be transferred to peroxide radicals with the formation of a resonance-stabilized tocopheroxyl radical [25,26]. Comparing the redox potentials to each other, *α*-tocopherol has the lowest one with 0.37 V in contrast to carotenoids which are around 1 V [27,28,29]. If the protective effect is analyzed in inhomogeneous solutions like liposomes, it results in different protective effects. Liposomes consist of uni-/multilamellar membranes and are therefore similar to the lipid bilayer of cells and a good model system to analyze the lipid oxidation in membranes [30]. Woodall et al., 1997, had analyzed the phospholipid hydroperoxide content in liposomes initiated by hydrophilic and lipophilic azo-compounds [31]. When initiating the radicals in the lipophilic phase, *α*-tocopherol had the strongest effect, followed by zeaxanthin, *β*-carotene, and lastly, lycopene. The polar antioxidants had a higher destruction rate than the non-polar ones. When initiating the radicals in the hydrophilic phase, the polar antioxidants still had a protective effect whereas the effect of *β*-carotene was slightly lower, and lycopene even had a pro-oxidative effect. Their destruction rates were, nevertheless, quite similar. The difference in their protective effect compared to the one in homogeneous solutions might be caused by their different orientation in the membrane (Figure 1) [32].

Due to their different polarity, the antioxidants are located in different places in the lipid bilayer. The non-polar ones are embedded within the membrane, whereas lutein spans the membrane vertically and thus borders to the aqueous phase, effecting the fluidity of the membrane [33,34]. The stiffening of the membrane reduces the amount of radicals penetrating it [35]. *α*-tocopherol is located vertically in the membrane due to its polar chroman ring and its non-polar phytol chain [36]. Another huge factor influencing the protective effect of carotenoids is the concentration of oxygen. Boehm et al., 2016, showed that lycopene in cells with zero percent oxygen has the highest protective effect which is strongly decreasing, almost to zero, by increasing the oxygen concentration [37]. Since there are some controversial effects known between the lipophilic antioxidants, and as the protective effect is often analyzed with chemical antioxidants, it is necessary to initiate the damage using the individual UV ranges in order to transfer the protective effect to UV-induced lipid oxidation damage. Thus, the harmful effects of the wavelength ranges we are exposed to can be investigated [7,8]. Further information can also be obtained on how to improve their effect via the regenerative effects of vitamin C [38,39,40]. Therefore, liposomes of soy lecithin, which contain phospholipids and also several unsaturated fatty acids, were produced and combined with different concentrations of antioxidants alone and/or as a two-component mixture [41]. The protective effect of the antioxidants, which includes the scavenging activity, was recalculated by quantifying the lipid peroxidation biomarker malondialdehyde (MDA) [42]. This in vitro study analyses the potential of individual natural antioxidants to protect lipids against UV-radiation-induced oxidation. Since more than 1000 carotenoids exist, three well-known carotenoids, lycopene, *β*-carotene, and lutein, were chosen for analysis [43]. Thus, a non-polar acyclic carotenoid can be compared to a non-polar but cyclic one and to a more polar cyclic carotenoid with a shorter chain of conjugated double bonds [44]. The selection of different polar antioxidants allows conclusions on the mechanism of antioxidant activity. The antioxidants selected in this study can be obtained from natural sources, like fruits and vegetables, enabling individuals to easily increase their intake of these antioxidants. Furthermore, the antioxidants can also be isolated from by-products of various food processing technologies, increasing sustainability.

## 2. Materials and Methods

### 2.1. Chemicals

The chemicals were of analytical-grade or HPLC-grade quality and, if not, otherwise specified from Merck KGaA (Darmstadt, Germany), VWR International GmbH (Darmstadt, Germany), Carl Roth GmbH + Co. KG (Karlsruhe, Germany) or Th. Geyer GmbH & Co. KG (Renningen, Germany). For the aqueous solutions, double-distilled water (18 MΩ) (Barnstead MicroPure UV system, Thermo Electron LED GmbH, Niederelbert, Germany) was used. The stock solutions of the lipophilic antioxidants, DL-*α*-tocopherol (>99%, Calbiochem-Merck Biosciences, Darmstadt, Germany), (*all-E*)-*β*-carotene (>93%, Sigma-Aldrich, Taufkirchen, Germany), (*all-E*)-lycopene, gift from Conesa (>99%, Villafranca del Guadiana, Spain) and (*all-E*)-lutein, extracted from an animal food additive (95% (*all-E*)-lutein, 5% Zeaxanthin; Gisis S.A., Guayaquil, Ecuador), were dissolved in dichloromethane. The internal standards for the quantification of the antioxidants were *β*-apo-8′-carotenal (>95%, CaroteNature, Münsingen, Switzerland) and *(all-rac)*-*α*-tocopheryl acetate (>96%, Sigma-Aldrich, Taufkirchen, Germany). The other carotenoid standards (97–99%) were also from CaroteNature (Münsingen, Switzerland). The concentration of the standards was determined using the Jasco V-530 UV/Vis-Spectrophotometer (Jasco, Pfungstadt, Germany). DL-*α*-tocopherol was diluted in ethanol and measured at 292 nm, (*all-E*)-*β*-carotene at 450 nm in n-hexane, (*all-E*)-lycopene at 470 nm in petroleum ether, and (*all-E*)-lutein at 445 nm in ethanol [45,46].

### 2.2. Liposome Preparation

The liposomes were produced by dissolving 250 mg soy lecithin in dichloromethane. The lipophilic antioxidants were added at this step. The solvent was evaporated using a rotary evaporator at 30 °C, and the residues were redissolved with an ultrasonic bath in 5 mL sodium phosphate (100 mmol/L, pH = 7.4) (Fluka Chemie AG, Buchs, Switzerland). If L(+)-ascorbic acid (≥99%, Th. Geyer GmbH & Co. KG) or 6-hydroxy-2,5,7,8-tetramethylchroman-2-carboxylic acid (Trolox, ≥96%, ThermoFisher GmbH, Kandel, Germany) was added to the liposomes, it was dissolved first in the buffer. The liposomes consisted of uni- and multilamellar vesicles [47].

### 2.3. Radiation

The liposome solution was irradiated for UV-C irradiation with 32.4 kJ/m^2^ for 3 min at 254 nm, for UV-B irradiation with 72.0 kJ/m^2^ for 20 min at 320 nm, and for UV-A irradiation with 144.0 kJ/m^2^ for 30 min at 365 nm in the UV-chamber M3 (Dinies Technologies GmbH, Villingendorf, Germany), in three Petri dishes standing on a cooling pad always at the same position. To calculate the influence of the UV irradiation alone, three samples were taken from the unirradiated solution and three from each Petri dish for the thiobarbituric acid-Assay (TBA-Assay). For the antioxidant degradation, one sample from each Petri dish was analyzed.

### 2.4. Quantification of Lipid Peroxidation: TBA-Assay

Lipid peroxidation was quantified by using the TBA-Assay. This assay uses MDA as a biomarker since it is produced during the lipid oxidation of polyunsaturated fatty acids [48]. MDA was derivatized with TBA to a pink dye, and to separate other thiobarbituric acid active substances, HPLC analysis was used [42].

The MDA calibration stock solution was produced by dissolving 1,1,3,3-tetramethoxypropane (98%, ThermoFisher GmbH) in 0.1 mol/L hydrochloric acid and incubated for 7 min at 95 °C (1000 rpm). The dilutions of the stock solution were made with water.

For the HPLC analysis, the samples were derivatized by adding 100 µL ethanol with 0.1% butylhydroxytoluene (BHT) (Sigma-Aldrich) and 600 µL 0.4% TBA, made by dissolving 2-thiobarbituric acid in 0.2 mol/L hydrochloric acid, to the 100 µL sample. The samples were incubated at 95 °C for 60 min (1000 rpm), cooled down on ice, and centrifuged (14,000 rpm, 5 min) prior to HPLC analysis [49].

For the quantification of MDA, a reversed-phase chromatography using a Merck Hitachi 7000 series HPLC system with a diode-array-detector (DAD) (Merck KGaA, Darmstadt, Germany and Hitachi Instruments, Inc., San Jose, CA, USA) was performed. The column (Luna^®^ C18, 250 × 4.6 mm, 5 µm, Phenomenex, Aschaffenburg, Germany) was tempered to 25 °C. The eluent was a mixture of potassium phosphate buffer (pH= 5.5)/methanol (55:45 [*v*:*v*]). The elution was isocratic with a flow rate of 0.75 mL/min for 12 min and a sample injection volume of 50 µL. The TBA-MDA adducts were detected after 7.9 min at 514 nm. Quantification was achieved with a 6-point calibration curve (concentration range: 0.21–4.26 mg MDA/mL, r > 0.999). The HPLC System Manager (Version 4.1, Merck KGaA, Darmstadt, Germany and Hitachi Instruments, Inc., San Jose, CA, USA) was applied for data evaluation.

### 2.5. Carotenoid Extraction and Analysis

Liposomes with a carotenoid content of 1 mg/g lecithin were made to analyze the carotenoid degradation in irradiated liposomes. Three samples of 500 µL unirradiated solution and three samples of 600 µL of the irradiated solution were taken for the extraction. The carotenoids were extracted by an exhausted extraction of three to four times for 15 min with methanol/tetrahydrofuran (1 + 1 [*v*:*v*] + 0.1% BHT (Sigma-Aldrich) by using an ultrasonic bath. Magnesium carbonate (≥40% Mg as MgO, Sigma-Aldrich), sodium sulfate, and glass beads (⌀ 4 mm) were added for a better extraction and *β*-apo-8′-carotenal as the internal standard. After each extraction, a centrifugation (5000 rpm, 5 min) followed to separate the phases. The combined extract solutions were evaporated using a rotary evaporator at 30 °C. The residue was redissolved in methanol/methyl tert-butyl ether (1 + 1 [*v*:*v*]) and centrifuged (14,000 rpm, 5 min) before HPLC analysis [50].

The carotenoids were analyzed using a VWR Hitachi Chromaster (5000 series) reversed-phase HPLC system (column: Develosil C30, 250 × 4.6 mm, 5 µm, Phenomenex) at a column temperature of 13 °C and 50 µL injection volume. An eluent gradient was applied at a flow rate of 1 mL/min. The gradient started with 90% (A) methanol and 10% (B) methyl tert-butyl ether; then, (B) increased to 50% in 40 min and, afterwards, to 60% in 2 min. This ratio was held for 23 min, before it turned back to the ratio of the start within 5 min and was lastly held for 5 min. For detection, a DAD was used at 450 nm. Evaluation was performed using the Chromaster system manager (Version 2.0, Hitachi High-Tech Science Corporation, Tokyo, Japan).

### 2.6. Tocopherol-Ectraction and Analysis

The tocopherol extraction was made as described for the carotenoids. The internal standard *(all-rac)*-α-tocopheryl acetate was taken instead and the residue was redissolved in n-hexane/methyl tert-butyl ether (98 + 2 [*v*:*m*]).

Vitamin E was analyzed by normal-phase chromatography using the Jasco LC-900 series HPLC system with a fluorescence detector (Eurospher-Diol column, 250 × 4.6 mm, 5 µm, Knauer Wissenschaftliche Geräte GmbH, Berlin, Germany) at a column temperature of 25 °C. The elution was made with n-hexane/methyl tert-butyl ether (98 + 2 [*v*:*m*]) isocratically with a flow rate of 1.5 mL/min for 40 min and an injection volume of 20 µL. For the fluorescence detector, the excitation wavelength was set to 292 nm and the emission wavelength to 330 nm. The data were evaluated by Jasco ChromNav (Version 1.18.07, Build 3) [51].

### 2.7. Ascorbic Acid Extraction and Analysis

For the ascorbic acid analysis, 200 µL of non-irradiated liposome solution or 400 µL of irradiated liposome solution were added to a 5 mL volumetric flask containing 20% *meta*-phosphoric acid and vortexed. This solution was filtered using syringe filters (syringe filters ROTILABO^®^ mixed cellulose ester (MCE), 0.2 µm, Carl Roth GmbH + Co. KG) and filled in vials for HPLC analysis. Syringe filters with mixed cellulose ester were chosen because they are suitable for most analytes [52].

For calibration, a stock solution of 1 mg/mL L(+)-ascorbic acid in 4.5% *meta*-phosphoric acid was prepared and diluted. Ascorbic acid was directly analyzed by reversed-phase chromatography using a Merck Hitachi 7000 series HPLC system with a DAD (Merck Hitachi). The column (Luna^®^ C18, 250 × 4.6 mm, 5 µm, Phenomenex) was tempered to 25 °C. The eluent was 0.5% acetic acid. For the calibration, an isocratic elution was used for 6 min and for the samples, a gradient with methanol was applied. The methanol content raised from zero to 100% within 4.5 min and held for 1 min before coming back to the initial conditions. The flow rate for both methods was 1.2 mL/min and the injection volume was 50 µL. The ascorbic acid was detected after 3.8 min at 254 nm.

### 2.8. Limits of Detection and Quantification

The limits of detection and quantification for each analyte were based for the antioxidants on the signal-to-noise ratios of S/N = 3:1 and S/N = 10:1 and for the MDA and ascorbic acid on the DIN 32645.

### 2.9. Statistical Analysis

**Figure 2:**
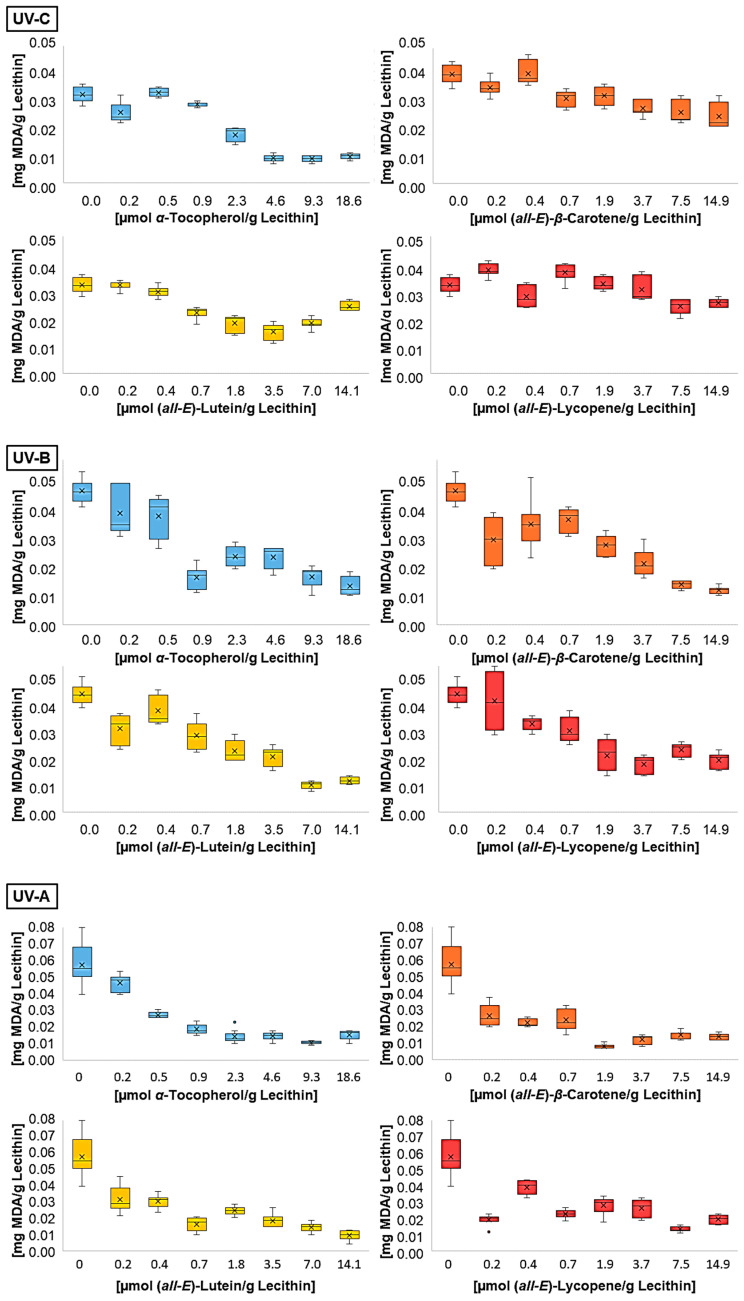
MDA concentrations after UV irradiation of liposomes containing different antioxidants at various concentrations (n = 9).

IBM^®^ SPSS^®^ Statistics (Version 27.0.0.0, Chicago, IL, USA) was used to define the significance of the results. One-factor ANOVA and Bonferroni or Games-Howell post hoc test (α = 0.05) were applied. Via the Levene’s test (*p* > 0.05), the homogeneity of variances was evaluated. The results are presented as mean ± standard deviation, except for Figure 2.

## 3. Results and Discussion

### 3.1. Protective Effects of Lipophilic Antioxidants in UV-Irradiated Liposomes

When selecting the irradiation time, several factors were considered. Firstly, the MDA concentration had to be sufficiently high in the liposome solutions without antioxidants so that an effect could be measured in the solutions with antioxidants. The duration of irradiation was chosen to be as short as possible in order to be closer to physiological conditions [8]. Since the irradiance of the lamps is significantly stronger than that of sunlight, it is difficult to transfer the measured results directly to natural conditions. Finally, the product of a radical chain reaction must be considered, so the scattering can vary at different times. Therefore, an irradiation time which causes the smallest scattering for the samples without antioxidant addition was chosen. When comparing MDA concentrations in the liposomes without additive after irradiation with different wavelengths, a correlation with a factor of 0.93 with the energy was observed (Figure 2). The analyzed UV ranges, dominated by different energy-rich wavelengths, showed no influence on the MDA content.

Figure 2 shows the MDA contents of liposomes containing different concentrations of lipophilic antioxidants after irradiation with the individual wavelengths. All antioxidants led to a reduction in the MDA concentration produced by UV radiation. The effect of the antioxidants decreased slightly with decreasing wavelength. In addition, the scattering of MDA concentrations was higher with smaller antioxidant levels. This is caused by the carotenoids, especially the used all-trans-isomers, which aggregate in inhomogeneous solutions such as liposomes [31,44,53]. The aggregation of antioxidants leads to an increase in the distance between radical and antioxidant and facilitates autoxidation within the liposomes. Distance and autoxidation are reduced by increasing the antioxidant concentration, leading to a lower scattering of MDA concentrations at higher levels of antioxidants. This is one possible reason for higher carotenoid concentrations in sun-exposed tissues like the forehead or the macula [14,54].

**Figure 3:**
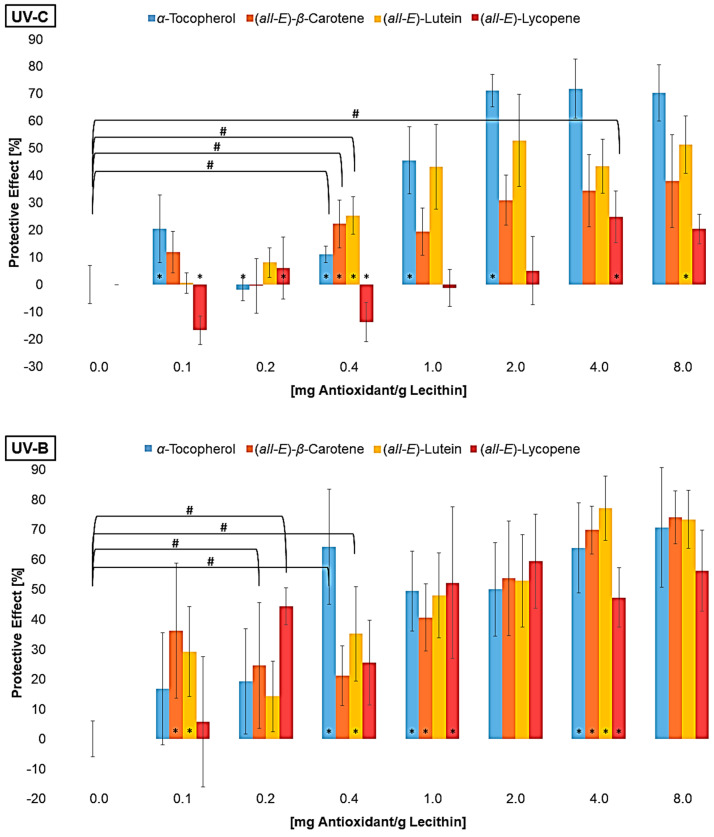

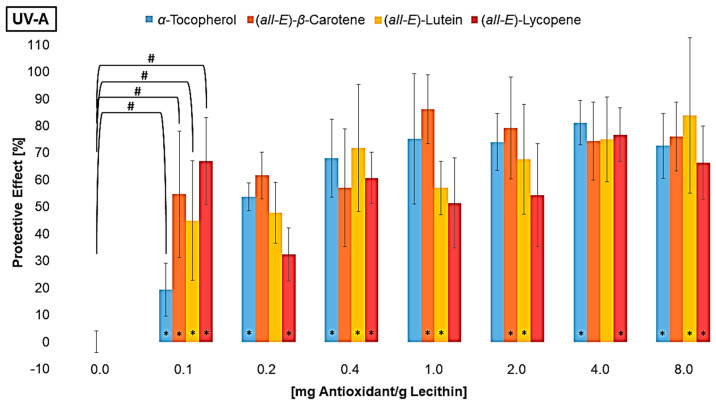
Protective effects of different antioxidants at various concentrations on lipid oxidation in liposomes, # = significantly protective compared to zero, * = significantly different to the next lower concentration, (n = 9, *p*-value < 0.05).

The MDA concentration of the liposomes with additive was converted to a protective effect compared to the control sample without additives (Figure 3).

To calculate the protective effect, the MDA concentration of the sample was subtracted from the control sample and set in relation to the control sample:

$$Protective\;Effect=\frac{control\;sample-sample}{control\;sample}\cdot 100$$

**Figure 4:**
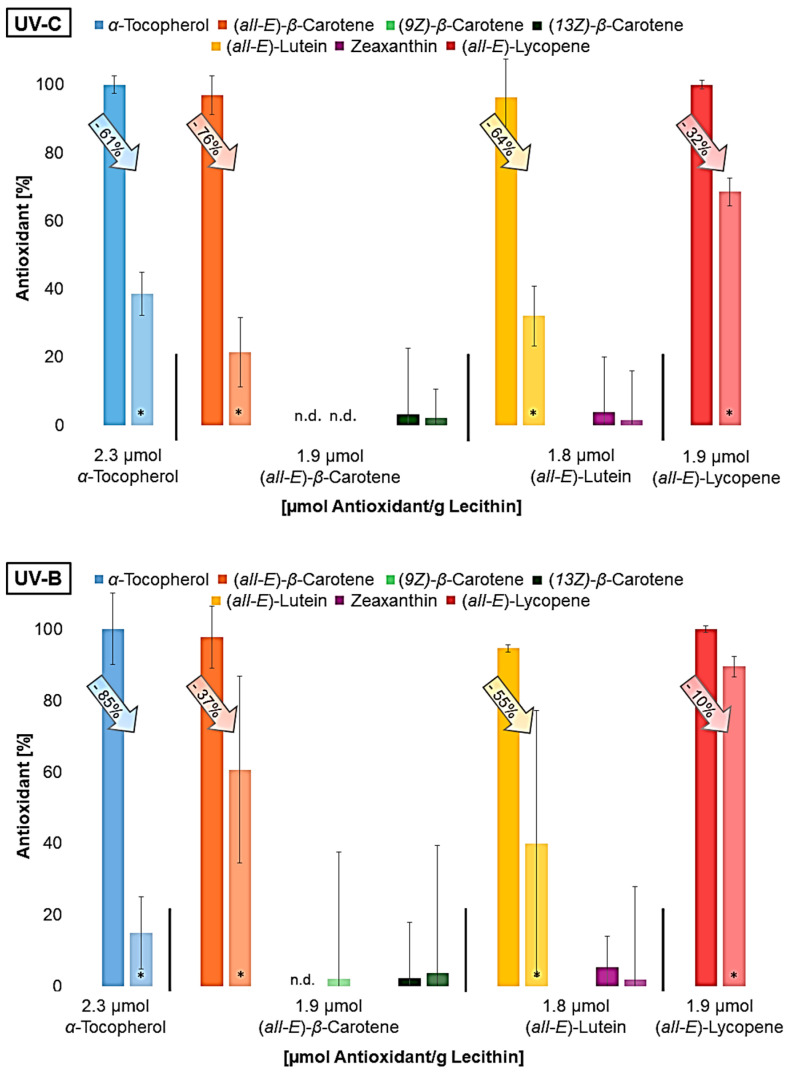

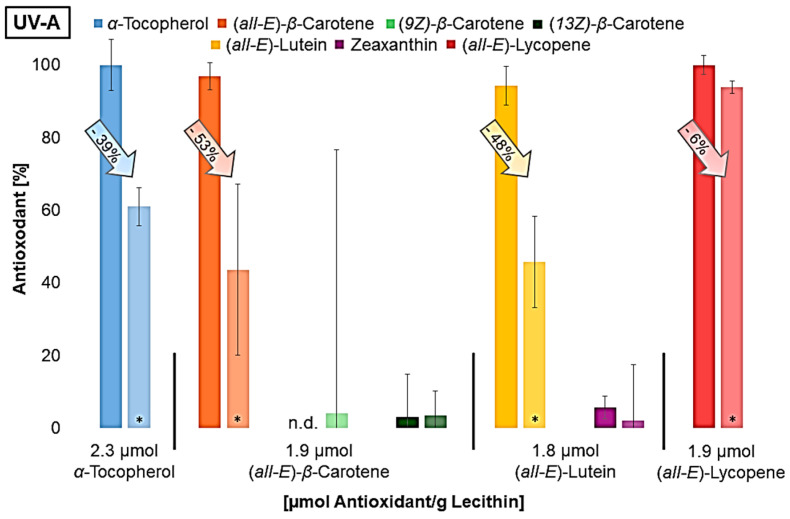
Antioxidant degradation in %, dark bar: non-irradiated liposome solution; lighter bar: irradiated liposome solution; n.d.= not detected; * = significantly different from the non-irradiated sample (n = 9, *p*-value < 0.05).

The differences in the scatter shown in Figure 2 are no longer present in Figure 3. On the contrary, the standard deviation tends to be higher for high antioxidant concentrations. This is due to the conversion of the data into percentage. Consistent scattering is higher at low MDA concentrations than at high concentrations. All three UV radiation ranges showed a non-linear increase and a stagnation in the protective effect of the individual antioxidants. The effect stagnates much faster under UV-A than under UV-B and UV-C radiation. Using UV-B or UV-C, it only stagnates at antioxidant concentrations that are close to saturation. The same was shown when looking at the first significant effect of an antioxidant. Under UV-A radiation, the lowest tested concentration of 0.1 µmol/g lecithin already showed an effect, while higher concentrations of antioxidants were required for protection against UV-B and UV-C. The antioxidant effect seems to correlate with the energy of the UV range and not with the energy of the radiation in total. Comparing the antioxidants, strong differences were found under UV-C radiation. The carotenoids had a higher protective effect with increasing polarity. The protective effect of lutein increased to 53%. In contrast, lycopene showed no effect or a pro-oxidant effect at low concentrations and an effect of 25% at higher concentrations. α-Tocopherol, which is even more polar than lutein but also absorbs the most UV-C radiation with an absorption maximum of 292 nm, showed the strongest effect of over 70% [46]. Under UV-B radiation, there were no significant differences between the strongest protective effects of α-tocopherol, lutein, and β-carotene. In contrast, lycopene showed a significantly poorer effect with only 55%. The stronger effect of β-carotene compared to lycopene could be due to the additional allylic hydrogen atoms on the ring. These are favored during hydrogen abstraction by radicals [55,56]. Under UV-A radiation, there were no differences between the antioxidants, with the effect of the individual antioxidants being around 80%. Factors influencing the protective effect emerging from these tests are the absorption spectrum, the polarity, and the redox potential. The latter is similar for the carotenoids (0.98–1.06 V) and 0.37 V for α-tocopherol [29,57]. The thermodynamic conditions for a redox reaction are therefore more favorable for α-tocopherol than for the carotenoids, which could mean that it tends to have a better effect. However, Furdak et al., 2025, showed that the comparison of redox potentials does not always provide a clear explanation for the results in antioxidant tests [28]. For example, the antioxidants used by Zanfini et al., 2010, were placed in the following order using the TEAC test (Trolox Equivalent Antioxidative Capacity): lycopene > β-carotene > lutein > α-tocopherol, which in turn would correspond to an opposite result [24]. The TEAC test showed a similar result to that of the quenching potentials. The quenching potential increases with the increase in the triplet energy level and, thus, the number of conjugated double bonds [58]. At higher wavelengths, the UV radiation experiments showed a comparable antioxidant activity for α-tocopherol and carotenoids, because the carotenoids can compensate for their lower redox potential by absorbing more UV-A radiation. For UV-B radiation, not being absorbed by any compound tested, the polarity and, therefore, the orientation in the lipid bilayer is of greater importance (Figure 1). α-tocopherol and lutein, which are located at the edge of the membrane and stiffen the membrane and scavenge radicals penetrating from the aqueous phase more rapidly, but also reduce the amount that can penetrate the membrane [35]. To obtain more information on the effects of the antioxidants, their degradation was examined (Figure 4).

**Figure 5:**
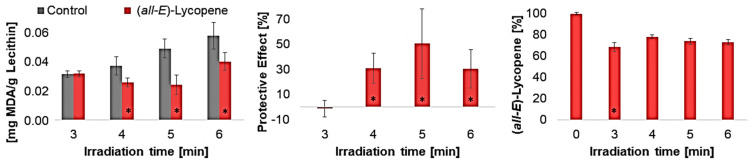
Influence of duration of UV-C radiation, **left**: MDA concentration in liposomes with and without 1.9 µmol lycopene/g lecithin, **middle**: protective effects of lycopene in liposomes, **right**: lycopene degradation in liposomes, * = significantly different from the shorter exposure time or control (n = 9, *p*-value < 0.05).

All antioxidants were degraded to a significant extent in UV-irradiated liposomes. *β*-carotene and lutein already contained different geometrical isomers prior to irradiation, as the used standards were not completely pure. It was not possible to determine whether the (*Z*)-isomers are increasingly formed due to irradiation. The isomers could have been formed by irradiation but have also been degraded. It can be assumed that the molecular structure has already changed significantly as a result of the irradiation, as typical degradation products of the carotenoids could not be detected using LC-MS. Shi et al., 2008, showed that, under daylight, (*all-E*)-lycopene is more stable than *cis*-isomers and can therefore lead to no or less *cis*-isomers, although they are firstly formed during the degradation of (*all-E*)-lycopene [59,60]. This will probably happen to the other carotenoids as well. Furthermore, antioxidant degradation showed similar results to the protective effect (Figure 3). This means that lycopene, the antioxidant that showed the least protective effect at all wavelengths, was also only minimally degraded compared to the other antioxidants. The degradation of the other antioxidants was very similar under UV-C and UV-A radiation; it increased only under UV-B radiation with enhanced polarity, which also correlates with the protective effect. The higher the antioxidant activity, the more it degraded. The result of the UV-A radiation was expected since the protective effect of the antioxidants was quite similar. Due to the low lycopene degradation observed, it was investigated whether lycopene shows a better effect than previously predicted by varying the irradiation time and thus the radiation energy (Figure 5).

The protective effect initially increased with increasing irradiation time, reaching its maximum at an irradiation time of 5 min, and then decreasing again. At an irradiation time of 6 min, many radicals were produced, leading to decreasing scavenging so that the protective effect decreased. Regarding lycopene degradation, there was no significant difference between the individual irradiation times. Consequently, the speed and quantity of radicals formed are crucial for the effect of lycopene.

Finally, the results showed that the effects of different antioxidants could potentially vary at other irradiation times and intensity.

### 3.2. Protective Effect of Vitamin C and Its Regenerative Effect on Lipophilic Antioxidants in UV-Irradiated Liposomes

**Figure 6:**
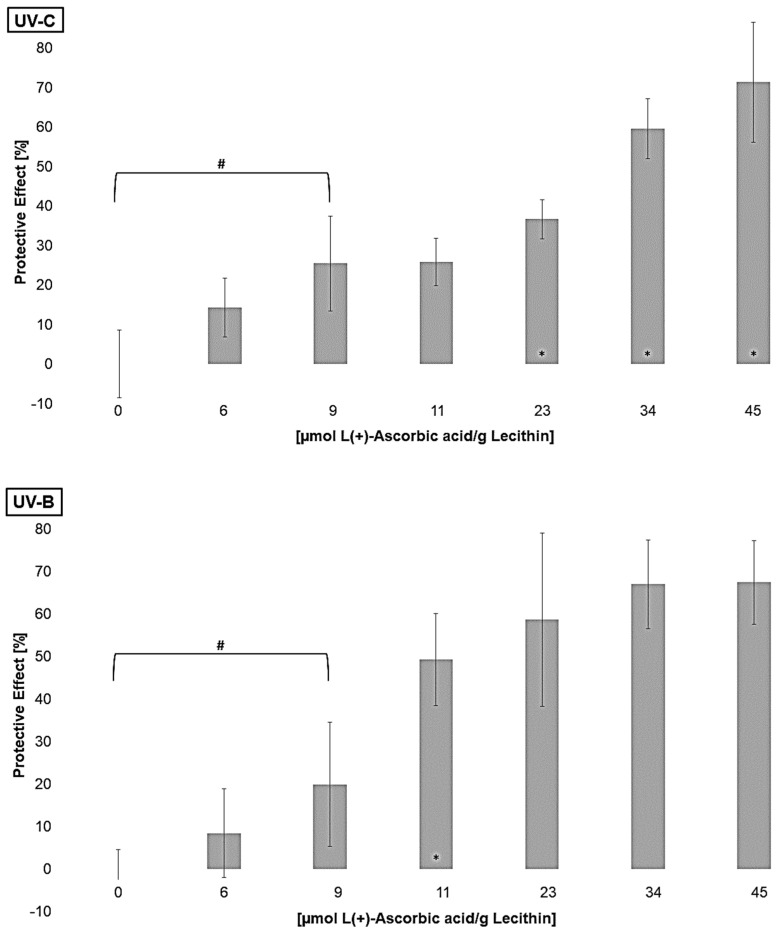

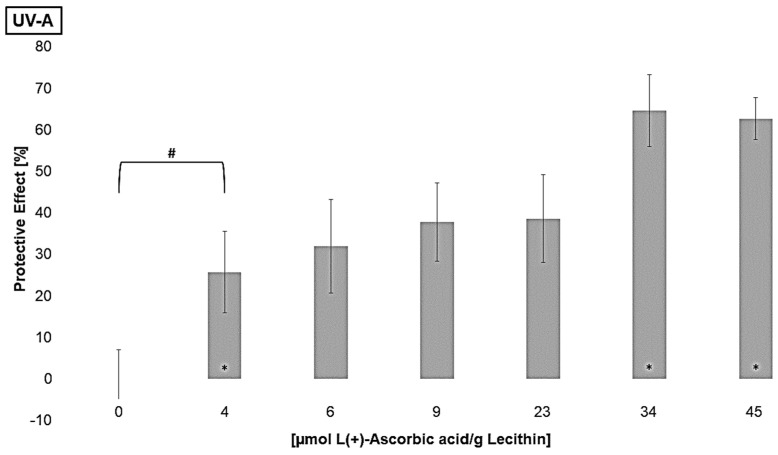
Protective effects of L(+)-ascorbic acid concentrations on lipid oxidation in liposomes, # = significantly protective compared to zero, * = significantly different to the next lower concentration, (n = 9, *p*-value < 0.05).

Furthermore, the regenerative effect of vitamin C on lipophilic antioxidants was investigated in order to analyze the enhancement of their effects [61]. Liposomes containing different concentrations of L(*+*)-ascorbic acid were irradiated to determine the lowest significantly effective concentration of ascorbic acid (Figure 6).

All tested concentrations showed either no effect or a protective effect. A pro-oxidative effect of vitamin C on its own, as Trommer et al., 2002, showed for UV-B irradiated stratum corneum lipid models, could not be observed [62]. A protective effect for UV-C and UV-B-irradiated liposomes was reached at a concentration of 9 µmol L(+)-ascorbic acid/g lecithin. For UV-A-exposed liposomes, a concentration of 4 µmol/g lecithin was already effective. The increasing antioxidant activity at higher wavelengths is thus comparable to the lipophilic antioxidants. The effect did not increase linearly and stagnated at around 70% under UV-A and UV-B radiation. Under UV-C radiation, the effect did not stagnate at the concentrations tested; presumably, even higher concentrations would lead to stagnation. Similar effects of vitamin C in comparison to *α*-tocopherol and *β*-carotene with unilamellar liposomes and the hydrophilic azo-compound radical initiator 2,2′-azobis(2-aminopropane)dihydrochloride were shown by Roberts and Gordon, 2003 [40]. Generating the radicals only in the aqueous phase, and although vitamin C is present in this phase, it does not show a better effect than if the radical generation takes place in both phases, even though its redox potential with 0.28 V is lower than the redox potentials of the other tested antioxidants [28].

**Figure 7:**
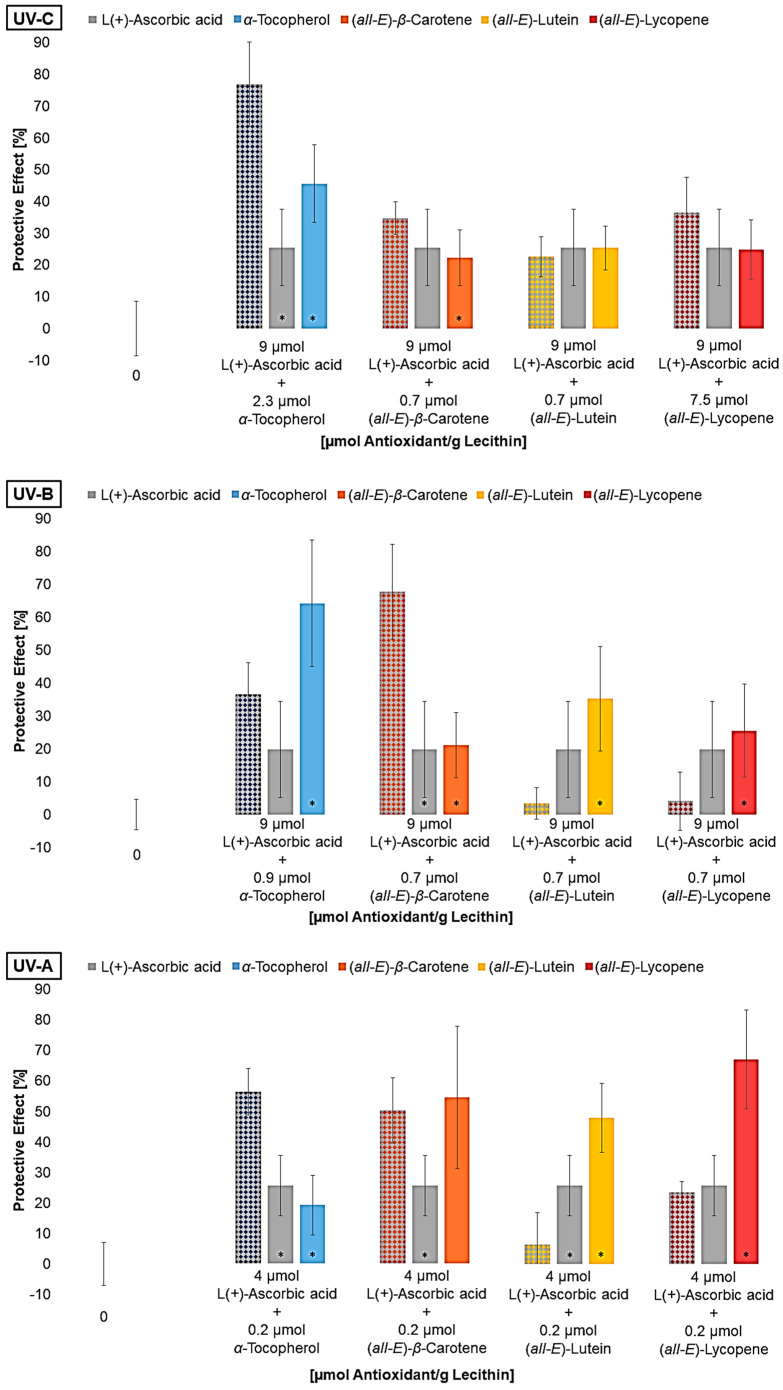
Protective effects of mixtures (first bar per group) of L(+)-ascorbic acid and different lipophilic antioxidants on lipid oxidation in liposomes, and the effects of the antioxidants alone shown alongside for comparison, * = significantly different to the mixture (n = 9, *p*-value < 0.05).

For the next experiments, liposomes were produced, each containing the lowest effective concentration of the lipophilic antioxidant and ascorbic acid as mixture (Figure 7).

Regarding the antioxidant mixtures, it must be pointed out that the presence of multilamellar membranes in liposomes makes it difficult to transfer the results to cell membranes. Considering individual antioxidants, this can be neglected as the antioxidant may or may not be close to lipids. The effect of the mixtures of ascorbic acid with lipophilic antioxidants was largely like the effect of the antioxidants alone. Only the mixture with *α*-tocopherol under UV-C and UV-A radiation and *β*-carotene under UV-B radiation showed the additive or synergistic effects of the antioxidants. Since vitamin C with a standard redox potential of 0.28 V represents a good redox system together with the lipophilic antioxidants, synergistic effects were also expected for the mixtures with lutein [19,28]. Lutein and *α*-tocopherol are located in the lipid bilayer due to the polar functional groups at the edge of the membrane [34,63]. As a result, these antioxidants can interact with ascorbic acid, whereas *β*-carotene is located in the membrane and, therefore, interactions are not expected. Consequently, the additive effect of this mixture can only be attributed to the fact that radicals and singlet oxygen were well scavenged in the aqueous and the lipophilic phase, thus enhancing the effect. Böhm et al., 1997, explained similar results by the assumption that the *β*-carotene radical is positively charged and thus can locate nearer to the edge of the membrane for interactions with ascorbic acid, leading to a synergistic effect [64]. The low protective effect of the mixture of lutein and vitamin C can be explained by the fact that radicals scavenged by lutein are stabilized in the membrane. Thus, lutein can act as a radical transfer bridge for radicals from the aqueous phase as Liang et al., 2009, predicted [35].

In addition to the pro-oxidative effect of vitamin C alone described by Trommer et al., 2002, his tested mixtures of *α*-tocopherol and ascorbic acid did not show any synergistic effect [62]. Consequently, it cannot be excluded that pro-oxidative effects were partly present in the mixtures, leading to the results shown.

### 3.3. Regenerative Effect in Mixtures of α-Tocopherol and Trolox

**Figure 8:**
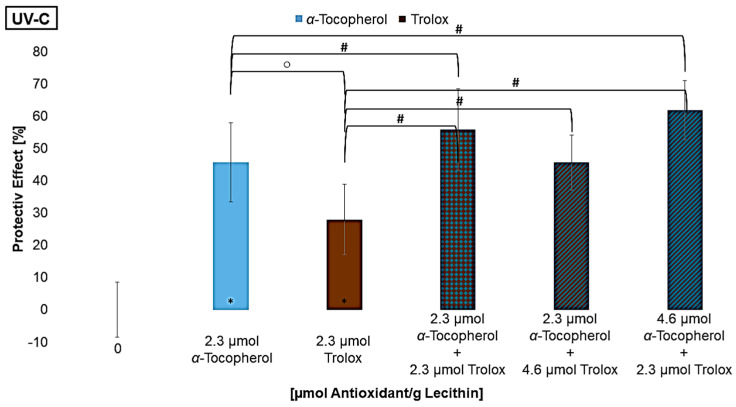

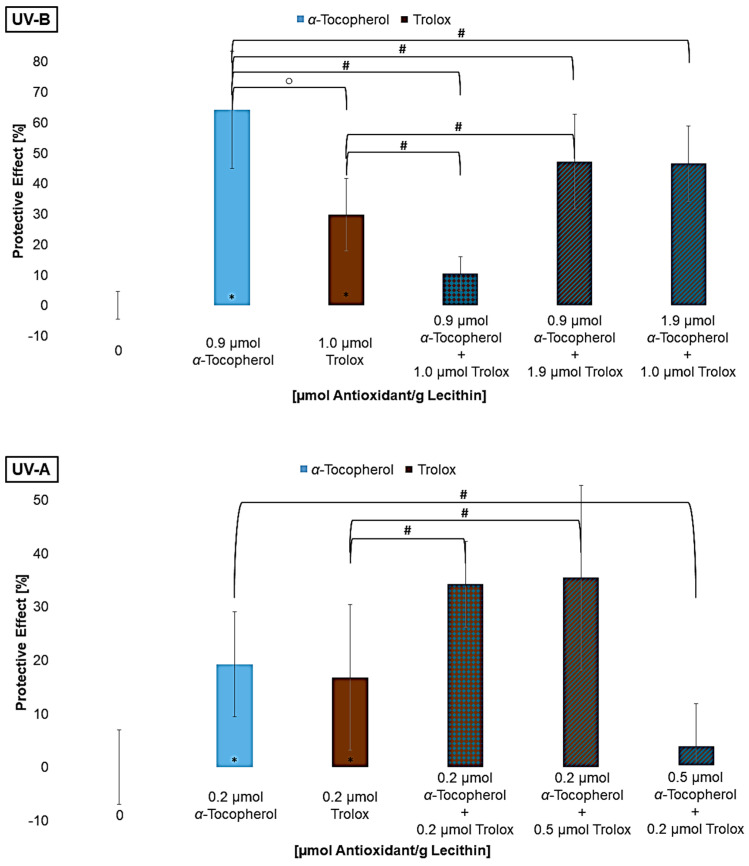
Protective effects of α-tocopherol and trolox, each alone as well as in mixtures on lipid oxidation in liposomes, ○ = significant difference between α-tocopherol and trolox, # = significantly different to the mixture, * = significantly different to the control (n = 9, *p*-value < 0.05).

To further analyze the mechanism of the regenerative effect, mixtures with different ratios of *α*-tocopherol and trolox were examined. Trolox is a vitamin E derivative which, compared to vitamin E, lacks the phytol chain, making trolox water-soluble. Thus, the interaction between two antioxidants that are present in different media but have the same mechanism of action can be compared (Figure 8) [65]. Compared to the mixtures with vitamin C, however, the redox potential of Trolox is higher than that of *α*-tocopherol. It can be assumed that the lipophilic antioxidant *α*-tocopherol, and not Trolox, regenerates the other antioxidant [28,57].

Using the same concentration as for *α*-tocopherol, trolox had a significantly smaller effect under UV-B and UV-C radiation, while no difference was observed under UV-A radiation. One reason can be that trolox is only present in the aqueous phase, so radicals within the lipid bilayer can no longer be scavenged before they react with an unsaturated fatty acid. Since trolox nevertheless showed a significant effect on its own, it can be assumed that the quenching of radicals in the aqueous phase effects protect the lipids in the bilayer. Lùcio et al., 2009, also found that the antioxidant activity of trolox and *α*-tocopherol is equally good if their localization in the membranes is considered for their protective effect [65]. If trolox is compared to vitamin C, trolox is the stronger antioxidant. The redox potential is not the reason for the effect, but rather the possibility of stabilizing the radical and making it less reactive. Since water-soluble antioxidants have a protective effect on lipid oxidation, the more polar lipophilic antioxidants have a stronger protective effect than the non-polar ones because their location at the edge of the membrane allows them to intercept radicals earlier. Thus, radicals are less able to penetrate deeply into the membrane [31,32,66]. The effect of the mixtures was significantly better, except the first one under UV-B radiation and the last one under UV-A radiation. However, varying the ratios by approximately doubling the concentrations of one component had no influence on the effect. In the mixture with a high level of *α*-tocopherol and a lower level of trolox under UV-A radiation, many *α*-tocopherol radicals may have been formed that were not regenerated by trolox, leading to a pro-oxidative effect. Additionally, since the antioxidant concentration is low, aggregation of the antioxidants may also have intensified this effect. Why one of the mixtures with α-tocopherol and trolox under UV-B radiation is not more protective than the antioxidants alone is not explainable. Nevertheless, it appears that specific concentrations are needed to have a synergistic effect when considering the results of Figure 7. Shi et al., 2004, analyzed two-component mixtures of lycopene with *β*-carotene, lutein, or *α*-tocopherol and also achieved a poorer effect in all mixtures compared to the antioxidants alone [67]. The same result that several mixtures do not achieve the expected protective effect was found by Chen et al., 2009, testing various mixtures with different concentrations of lycopene, *β*-carotene, and vitamin E and C [38].

## 4. Conclusions

The present results show that the localization of antioxidants has a strong influence on their protective effect. As the antioxidants are located at the edge of the membrane, they can scavenge radicals in the lipophilic phase, and radicals of the aqueous phase penetrate less deeply in the membrane. Also, xanthophylls, which span through the membrane, can function as a radical transfer bridge, causing a pro-oxidative effect in mixtures. Along with the scavenging capacity, the absorption of the wavelength has a remarkable influence, shown by *α*-tocopherol with its stronger protective effect compared to the carotenoids under UV-C radiation versus UV-A radiation.

Comparing the tested concentrations with serum concentrations, the lowest concentration of the carotenoids is 20–100 times larger and for *α*-tocopherol, 2–3 times lower [11,68,69]. So, in the human body, *α*-tocopherol might be of higher importance for the protection against lipid oxidation than the carotenoids. However, the carotenoid concentrations tested strongly differ from those in skin [54]. The energy used for irradiation was significantly stronger than that of which humans are exposed to. Thus, the present experiments needed a higher antioxidant concentration.

Finally, the examination of antioxidant mixtures in model systems appears to be challenging, as pro-oxidative effects are often observed. Since the concentration of antioxidants is not affected by other metabolic reactions in the model systems, as within the human body, non-physiological concentrations or ratios were investigated. The next step in analyzing the protective effect of antioxidants against skin cancer is the use of in vitro skin models, which will allow further insight into the mechanisms of action while still examining the effects of individual antioxidants.

## Data Availability

The original contributions presented in this study are included in the article. Further inquiries can be directed to the corresponding author.

## Abbreviations

The following abbreviations are used in this manuscript:

DAD	Diode array-detector
HPLC	High-pressure liquid chromatography
MDA	Malondialdehyde
n.d.	not detected
UV	Ultraviolet
TBA	Thiobarbituric acid
TEAC	Trolox Equivalent Antioxidant Capacity
